# Functional Study of *SAMD9L* in Familial Gastric Cancer

**DOI:** 10.5152/tjg.2023.22267

**Published:** 2023-05-01

**Authors:** Xu Kaixuan, Zhang Xiaobin, Tang Jiaxuan, Liu Shihui, Wang Xinxin, Hu Shuwei, Dai Penggao, Luo Xiang

**Affiliations:** 1 National Engineering Research Center for Miniaturized Detection Systems, Northwest University College of Life Sciences, Xi’an, China; 2 Shaanxi Lifegen Co., Ltd, Xi’an, China; 3 The University Hospital of Northwest University, Xi’an, China; 4 Department of Respiratory, Tongchuan People’s Hospital, Tongchuan, China

**Keywords:** Familial gastric cancer, SAMD9L, susceptibility gene, whole-exome sequencing

## Abstract

**Background:**

Familial aggregation occurs in approximately 10% of cases of gastric cancer. The genetic predisposition or cause of the disease in only about 40% of hereditary gastric cancer cases is known, while the genetic factors of the remaining cases remain to be studied.

**Methods:**

Samples were collected from a family with gastric cancer, including 3 gastric cancer and 17 healthy samples. Whole-exome sequencing was performed on samples from 3 patients with gastric cancer and 1 sample from healthy peripheral blood. *SAMD9L* was knocked down using small interfering RNAs and short hairpin RNA. The expression of *SAMD9L* was detected by quantitative real-time polymerase chain reaction and Western blot in SGC-7901 cells. CCK-8 assay was used to detect the proliferation of gastric cancer cells. The migration and invasion of gastric cancer cells were detected by Transwell assay and scratch assay. The cell apoptosis was detected by flow cytometry.

**Results:**

Twelve single-nucleotide variants and 9 insertions/deletions mutation sites were identified as candidate genes. Among them, *SAMD9L* regulates cell proliferation as a tumor suppressor gene. The experiments of knocking down *SAMD9L* in SGC-7901 cells revealed that reduced expression of *SAMD9L* significantly enhanced the proliferation, migration, and invasion of SGC-7901 cells.

**Conclusions:**

These results suggest that *SAMD9L* inhibits the proliferation of gastric cancer cells, thereby increasing the risk of gastric cancer in people with *SAMD9L* downregulation. Therefore, *SAMD9L* may represent a susceptibility gene of this gastric cancer family.

**Main Points**

- Samples were collected from a family with gastric cancer, including 3 gastric cancer and 17 healthy samples. Then, whole-exome sequencing was performed on 3 cases of gastric cancer and 1 healthy peripheral blood sample.
- The *SAMD9L* gene is a candidate susceptibility gene and the p.T832FS mutation causes premature truncation of *SAMD9L*. This mutation, described here for the first time to our knowledge, was further analyzed for its effects on cell function.
- The *SAMD9L* gene exerted inhibitory effects on cell proliferation, migration, and invasion.

## INTRODUCTION

Gastric cancer is one of the most common gastrointestinal malignancies and the third leading cause of cancer death worldwide.^1^ Particularly in certain countries in East Asia, Central and Eastern Europe, and Central and South America, the incidences and mortalities of gastric cancer are very high.^2^ Risk factors for gastric cancer include *Helicobacter pylori* infection, tobacco and alcohol use, lack of dietary fresh fruits and vegetables, consumption of processed meats, and obesity.^3,4^ The main molecular mechanisms of gastric cancer include key gene mutations, epigenetic changes, and noncoding RNAs.^5^ The sequence length of the exon region of the human genome is approximately 1% of the genome, but it contains >85% of known deleterious mutations that cause structural and functional abnormalities in proteins, leading to the development of related diseases.^6,7^ For example, *TP53* is the most frequently mutated key driver gene in gastric cancer, which leads to the loss of a cell’s DNA damage detection function, which may cause abnormal cell growth.^8^ Harmful mutations occur in a large number of protein-coding genes in gastric cancer, such as in *DCC* and APC/MCC, which are common in patients with early gastric cancer.^9-11^ Further, *APC* mutations render its negative regulation of the β-catenin receptor ineffective, leading to continuous activation of the Wnt signaling pathway.^12^

Although most gastric cancers are sporadic, approximately 10% of cases are present as familial clusters, possibly caused by heritable pathogenic mutations that increase the risk of gastric cancer. Hereditary diffuse gastric cancer (HDGC) confers autosomal-dominant genetic susceptibility to cancer syndrome, among which approximately 40% are caused by pathogenic mutations of *CDH1*. Although numerous patients do not harbor *CDH1* mutations, single allele expression is downregulated, which plays an important role in cell polarity and adhesion.^13^ Further, recent studies show that a *CTNNA1* lineage mutation is the genetic cause of the disease.^14^ The main clinical methods for treating familial gastric cancer (FIGC) are prophylactic gastrectomy and endoscopy, although potential therapeutic drugs remain to be developed.^15^ Gastric adenocarcinoma and proximal polyposis of the stomach (Gapps) is incompletely characterized, and some carriers may have normal endoscopy findings. However, there are few relevant studies, and the genetic cause of its association with FIGC is unclear.^16^ Sterile Alpha Motif Domain-containing 9 Like (*SAMD9L*) is tumor suppressor gene located on human 7q21, adjacent to its homologous gene, *SAMD9*, in a head-to-tail orientation.^17^ Natural mutations in the *SAMD9L* gene are associated with diseases such as myelodysplastic syndrome, acute myeloid leukemia, and ataxia-pancytopenia syndrome.^18-21^ Recently, frameshift mutations in *SAMD9L* have been found to be associated with systemic autoinflammatory disease and show a GoF in restricting cell growth.^22,23^

Here we collected peripheral blood samples from members of a family with gastric cancer. We performed full exome sequencing of 3 gastric cancer cases and 1 healthy sample. High-quality data for harmful mutated genes were obtained through bioinformatics analysis, candidate susceptibility genes were screened according to the family lineage, and mutation sites were verified using Sanger sequencing. *SAMD9L*, which may be associated with gastric cancer, was selected for functional experiments according to literature reports and database analysis.^24,25^

## MATERIALS AND METHODS

### Clinical Peripheral Blood Samples and Cell Culture

Peripheral blood samples were collected at the First Affiliated Hospital of Xi’an Jiaotong University from 3 cases of gastric cancer and 17 healthy samples in a family with gastric cancer. The protocol of this study was approved by the Ethics Committee of the First Affiliated Hospital of Xi‘an Jiaotong University, and written informed consent was acquired from all recruited patients. The human GC cell line SGC-7901 was obtained from the Cell Bank of the Chinese Academy of Science (Shanghai, China). Cells were cultured in Dulbecco’s Modified Eagle’s Medium (DMEM) medium (Procell Life Science & Technology Co., Ltd.) supplemented with 10% fetal bovine serum (Gibco, Carlsbad, Calif, USA) and 1% antibiotics (100 U/mL penicillin G and 100 mg/mL streptomycin; Gibco, Carlsbad, Calif, USA).

### Whole-Exome Sequencing

DNA was extracted from peripheral blood samples using an EZNA Blood DNA Kit (Omega Bio-tek) following the manufacturer’s instructions. The sequencing was performed on peripheral blood DNA from individuals II-3, III-1, and III-2 from the family using Illumina HiSeq2000.

### Sanger Sequencing

Capillary sequencing using customized primers was performed to verify the reliability of whole-exome sequencing. Primer sequences are shown in Table 1. Sangon Biotech (Sangon, Shanghai, China) performed sequencing of the DNA fragments.

### RNA Isolation and Quantitative Real-Time Polymerase Chain Reaction

The extraction of total RNA from cell lines was performed following protocols provided by the manufacturer (TRIzol reagent, Invitrogen, Carlsbad, CA, USA). Total RNA was quantified using a Nanodrop ND-1000 spectrophotometer (Nanodrop Technologies; Thermo Fisher Scientific, Inc., Wilmington, Del, USA). To detect gene expression, the samples were amplified using an ABI ViiATM 7 with a One Step SYBR PrimeScript™ RT-PCR Kit II (Takara Bio, Japan) according to the manufacturer’s instructions. The data were normalized to the levels of the mRNA-encoding glyceraldehyde 3-phosphate dehydrogenase (*GAPDH*). Relative expression levels of target genes were determined via the 2^−ΔΔCT^ method. Primers specific for *SAMD9L*, *SAMD9L*-Mutant, and *GAPDH* are listed in Table 2.

### Transfection

Chemically synthesized *SAMD9L* small interfering RNAs (siRNAs), specific short hairpin RNA (shRNA), and the *SAMD9L* p.T832FS plasmid expression vector pEX-3-mutant were from Gene Pharma Company (Shanghai, China). Transfections were performed using Lipofectamine 2000 reagent (Invitrogen) in 6-well plates according to the manufacturer’s instructions. The targeting sequences were listed in Table 3.

### Cell Proliferation Assay

Transfected cells were digested, collected, and added to the wells of 96-well plates at an initial density of 2 × 10^3^ cells per well. After cells were completely attached to the well, the medium in the first column of the 96-well plate was discarded. Cell viability was determined using CCK-8 reagent (CK04-100, Dojindo, Kumamoto Prefecture, Kyushu, Japan) according to the manufacturer’s protocols.

### Colony Formation

Transfected SGC-7901 cells undergoing exponential growth were trypsinized, harvested, and added to 6-well plates, which were incubated for 2 weeks. When a single cell colony contained ≥50 cells, the 6-well plates were removed for staining. After washing in PBS, colonies containing >50 cells were fixed in methanol (Beyotime, Shanghai) and stained with crystal violet (Solarbio, Beijing). After the 6-well plates were completely dry, photos were taken.

### Cell Migration and Invasion Assays

Transfected cells were digested and collected, and a cell suspension was prepared in basal medium. Then 5 × 10^3^ cells were added to a well of a 24-well Transwell Boyden chamber (Corning R, Corning, Ny, USA). Cells were resuspended in 100 μL of complete medium containing 10% fetal bovine serum (FBS) in the upper chamber, and the lower chamber was filled with 0.6 mL of complete medium containing 20% FBS. After incubation for 12 hours, cotton swabs were used to remove cells from the upper chamber, and the remaining cells were fixed with 4% paraformaldehyde for approximately 30 minutes and stained with 0.1% crystal violet for 10 minutes. The number of migrated cells was counted in 5 random microscope fields using ImageJ. For cell invasion assays, 0.05 mL of Matrigel (50 μg/mL, BD Biosciences, San Jose, Calif, USA) was added to the plate’s surface, and cells were incubated for approximately after 3-5 hours at 37°C. The plates were treated as described above.

### Wound-Healing Test

Cells were added to 6-well plates, and the density of transfected cells reached approximately 80%. Then, slowly scratch the monolayer using a 200-μL pipette tip across the center of the well. The cells were subsequently placed in an incubator. Photographs were taken every 24 hours for the next 2 days. The cells were washed before photography to remove impurities and floating cells, and the medium was replaced with a new basic culture medium.

### Apoptosis Assay

Transfected cells were digested and resuspended in binding buffer and stained with fluorescein isothiocyanate-conjugated Annexin V together with propidium iodide (Keygen Biotech, Jiangsu, China). Apoptotic cells were detected using flow cytometry.

### Western Blot Analysis

Total proteins were extracted using radioimmunoprecipitation assay (RIPA) buffer (Beyotime, Shanghai, China) supplemented with protease inhibitors. Then, the protein was separated on sodium dodecyl sulfate-polyacrylamide gel electrophoresis (SDS-PAGE) gels and transferred to polyvinylidene fluoride (PVDF) membranes (Millipore, Bedford, MA, USA). PVDF membranes were incubated with primary antibody (anti-SAMD9L, 1:800 dilution, ZenBio, 862155) and incubated overnight at 4°C. Then, after adequate washing in Tris-HCl (pH 7.6) buffer containing the surfactant 0.1% Tween 20 and a 1-hour incubation with a second antibody (Beyotime, Shanghai, China), finally, the band density was analyzed with an ECL detection system and quantified using ImageJ software (National Institutes of Health, USA).

### Statistical Analysis

Data were analyzed using Statistical Package for Social Sciences software (version 18.0; SPSS Inc.; Chicago, IL, USA) and GraphPad Prism software (version 5.01, La Jolla, California, USA). The overall survival was plotted using the Kaplan–Meier method. Data are expressed as the mean ± SD. *P* < .05 was considered statistically significant.

## RESULTS

### Family Samples of Gastric Cancer and Whole-Exome Sequencing

**Figure 1.**
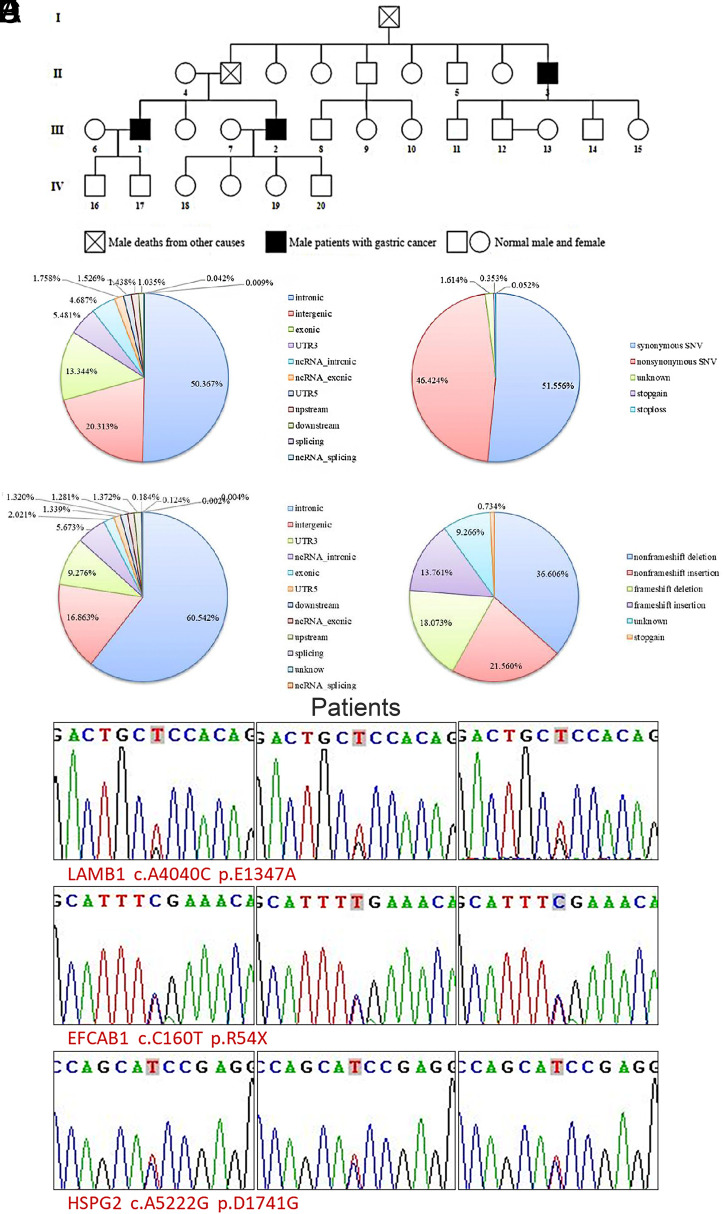
Family samples of gastric cancer and whole-exome sequencing. (A) Distribution of SNVs in different regions of the genome. (B) Statistical analysis of SNVs variants in the coding region. (C) Distribution of InDels in different regions of the genome. (D) Statistical analysis of InDels variants in the coding region. (E) Sanger sequencing results. (F) Genogram of a gastric cancer family. InDels, insertion and deletion sites; SNVs, single-nucleotide variants.

In the present study, we collected samples from members of a family with gastric cancer. There were 4 generations in this family, and 3 males suffered from gastric cancer (Figure 1A). Whole-exome sequencing was performed on 3 cases of gastric cancer and 1 healthy peripheral blood sample from this family. A total of 129 526 single-nucleotide variant (SNV) mutation sites were obtained, of which 17 284 were located in exons, among which 8024 were nonsynonymous mutations (Figure 1B and 1C). A total of 53 936 insertions/deletions (InDels) mutation sites were detected, of which 1090 were located in exons, including 347 frameshift mutations predicted to alter the open reading frame, leading to abnormal protein function (Figure 1D and 1E). To verify the reliability of whole-exome sequencing results, 3 candidate genes were randomly selected, and specific primers were designed to identify mutation sites. The results show that identical mutations were identified using whole-exome and first-generation sequencing (Figure 1F).

Key susceptibility genes were screened as follows: (1) the mutation located in the region of an exon or splice site, (2) nonsynonymous and frameshift mutations and gain or loss of a termination codon, (3) allele frequencies of the mutated loci in the database <1%, and (4) heterozygous mutations in patients’ samples and no mutation in the healthy sample (No. 4). Totally, 240 SNVs mutation sites and 47 InDels mutation sites were identified. Further, the prediction of amino acid structure indicates the influence of an amino acid substitution caused by a mutation on protein structure and function. The predicted results were judged deleterious or potentially deleterious, and the mutation sites with heterozygous mutations in the 3 gastric cancer samples were selected as representatives of candidate susceptibility genes. Twelve SNVs mutation sites and nine InDels mutation sites were detected (Table 4).

### Screening for Susceptibility Genes and Bioinformatics Analysis

**Figure 2.**
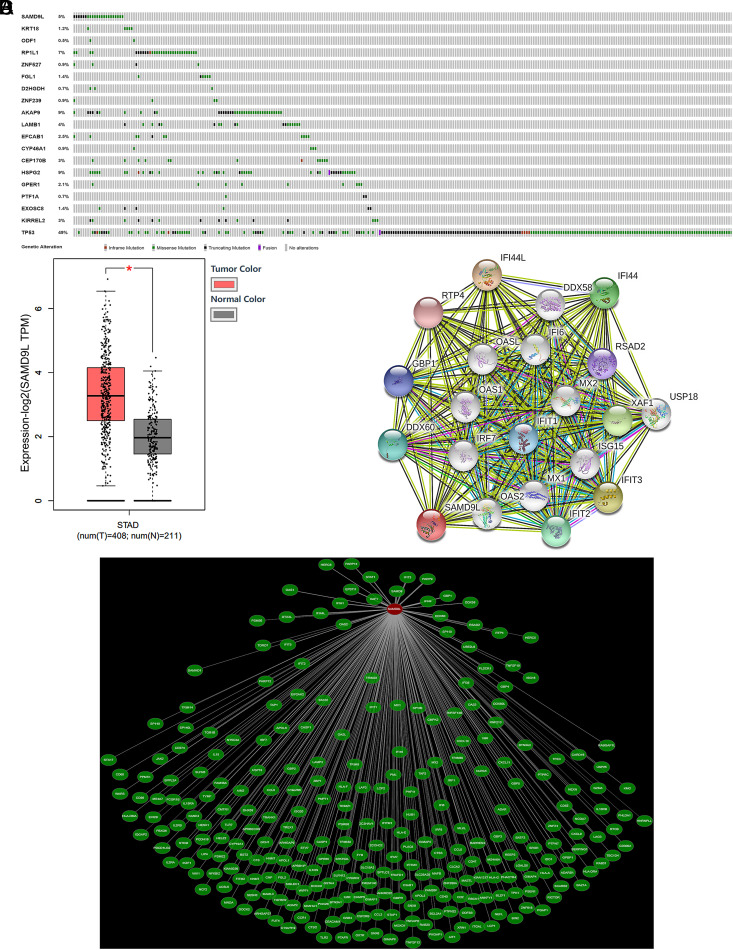
Screening for susceptibility genes and bioinformatics analysis. (A) Expression levels of *SAMD9L* in GC (n = 408) and normal epithelium tissues (n = 211) based on The Cancer Genome Atlas data. (B) Mutation frequency of candidate genes in TCGA gastric cancer samples. (C) Coexpression analysis of the *SAMD9L* gene, where the length of the line between the relevant gene and the *SAMD9L* gene represents edges’ LLS (log-likelihood score) score, reflecting the intensity of a correlation. (D) SAMD9L protein interaction network. GC, gastric cancer; TCGA, The Cancer Genome Atlas.

The cBioPortal (http://www.cbioportal.org/) was used to analyze the mutation frequencies of candidate genes in gastric cancer samples included in The Cancer Genome Atlas (TCGA) data. The tumor suppressor gene *P53* is mutated and inactivated in diverse malignant tumors, which may cause abnormal cell proliferation. The mutation frequency of *TP53* in gastric cancer samples in TCGA data is as high as 49%. The mutation frequency of the *SAMD9L* gene in gastric cancer samples was 5%, higher than other candidate susceptibility genes, suggesting that it may be closely associated with gastric cancer (Figure 2A). Next, analysis of TCGA clinical data revealed that *SAMD9L* gene expression is downregulated in gastric cancer tissues compared with normal tissues (http://gepia.cancer-pku.cn/index.html) (Figure 2B). Further, several studies showed that *SAMD9L* plays an important role in the regulation of cell proliferation as a tumor suppressor.^24,25^ Furthermore, we constructed a SAMD9L protein interaction network utilizing the Coexpedia (http://www.coexpedia.org/) and STRING (https://string-db.org/) websites, as depicted in Figure 2C and 2D. The results encouraged us to identify the function of the *SAMD9L* gene in gastric cancer.

### Knockdown of *SAMD9L* Promotes the Proliferation, Migration, and Invasion of Gastric Cancer Cells

**Figure 3.**
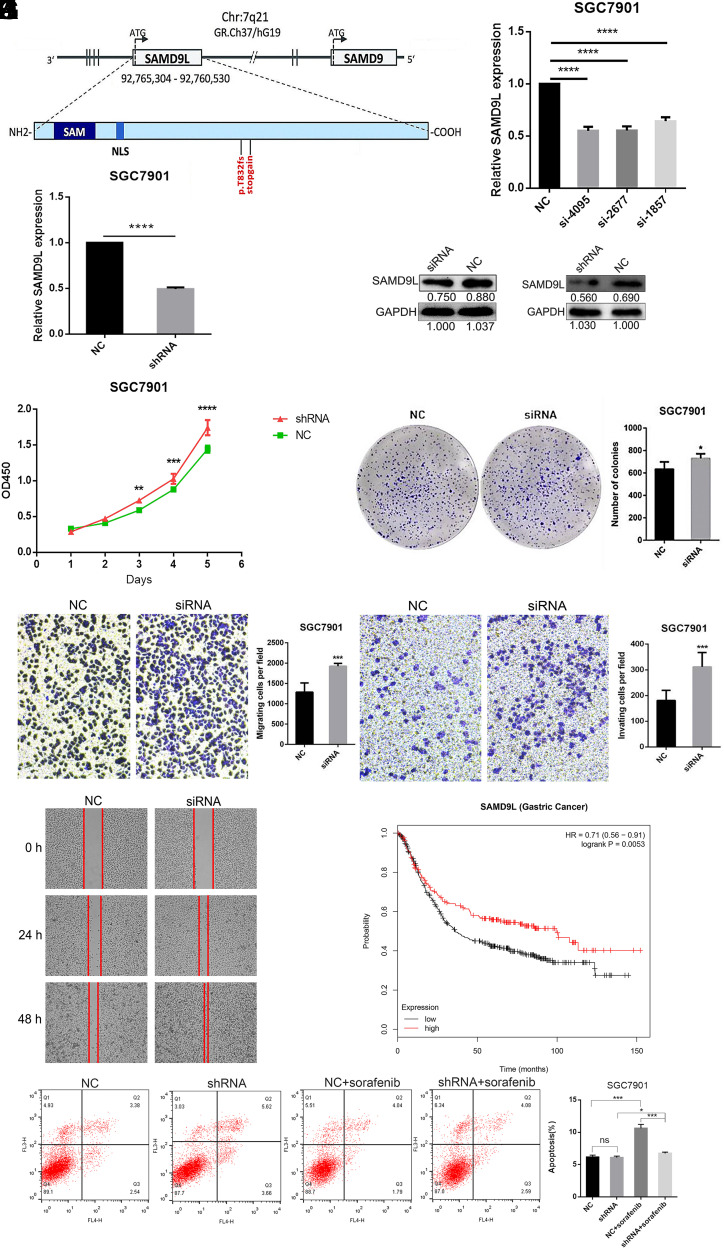
Knockdown of *SAMD9L* promotes gastric cancer cell proliferation, migration, and invasion. (A) *SAMD9L* gene structure and mutations. (B, C) *SAMD9L* knockdown effect was verified using siRNA and shRNA by qRT-PCR. (D) Western blot indicated knockdown effect of SAMD9L using siRNA and shRNA. (E) Knockdown of *SAMD9L* promotes gastric cancer cells to proliferate. (F) *SAMD9L* knockdown increased the colony-forming ability of SGC-7901 cells. (G, H) *SAMD9L* knockdown enhances migration and invasion of SGC-7901 cells. (I) Migration of SGC-7901 cells at different times after *SAMD9L* knockdown. (J) Survival as a function of SAMD9L expression levels in gastric cancer. (K) Effects of *SAMD9L* gene knockdown on sorafenib-induced apoptosis in SGC-7901 cells. The error bars represent the standard deviation (n = 3). N.S. = *P* > .05, ^*^P < .05, ^**^P < .01, ^***^P < .001, and ^****^P < .0001. shRNA, short hairpin RNA; qRT-PCR, quantitative real-time polymerase chain reaction.

The mutation p.T832fs of *SAMD9L* carried by patients in this gastric cancer family was caused by the insertion of a T base in the coding sequence corresponding to amino acid residue 832, which introduces a frameshift mutation. Interestingly, the mutation indirectly generates a stop codon at amino acid residue 847. This mutation results in the truncation of approximately 50% of the coding sequence of *SAMD9L* (Figure 3A).

Three *SAMD9L*-specific siRNA and shRNA sequences were used to transfect SGC-7901 cells, and those with the highest interference efficiency were selected for subsequent experiments (Figure 3B and 3C). Furthermore, Western blot analysis revealed that the protein level of *SAMD9L* was reduced (Figure 3D). Knockdown of *SAMD9L* enhanced the proliferative capacity of cells compared with the negative control (Figure 3E). A colony formation assay showed that *SAMD9L*-knockdown cells had a stronger ability to form colonies compared with the negative control cells (Figure 3F). The migration and invasion of SGC-7901 cells were significantly enhanced by downregulation of *SAMD9L* (Figure 3G and 3H). These data are consistent with those of a wound-healing assay, in which the wound closure rate was significantly increased in *SAMD9L* knockdown SGC-7901 cells (Figure 3I). Flow cytometry experiment showed that the 20-μM sorafenib-induced apoptosis was rescued by *SAMD9L*-knockdown in SGC-7901 cells (Figure 3K).

The relationship between the expression levels of *SAMD9L* and the survival of patients with gastric cancer was analyzed using the Kaplan–Meier Plotter website (http://www.kmplot.com/analysis/index.php?p=service&cancer=gastric). As predicted, patients with high expression levels survived longer, with a median survival of 99.4 months compared with 36.4 months for those with low expression levels (Figure 3J). To conclude, these findings suggest that downregulation of *SAMD9L* stimulates the proliferation, migration, invasion, rescues sorafenib-induced apoptosis, and was associated with poor prognosis.

## DISCUSSION

In this study, a new mutation site, *SAMD9L* p.T832fs, discovered here, arose because of an insertion of a T base at base 2496 of its coding region, resulting in a frameshift mutation of the protein SAMD9L after amino acid residue 832. The encoded protein most likely possesses an abnormal structure and function. Here we found that restrained expression of SAMD9L protein enhanced the proliferation, colony formation, migration, and invasion of SGC-7901 cells, and rescued sorafenib-induced apoptosis. Considering recent studies that *SAMD9L* gain-of-function variants inhibit global protein synthesis, reduce translation elongation, and induce proteotoxic stress response.^23,26,27^ Our findings preliminarily reflect *SAMD9L* as a tumor suppressor gene and may be closely related to the potential function of the mutant of *SAMD9L*.

The mutation frequency of candidate susceptibility genes in gastric cancer samples in TCGA data was subjected to bioinformatics analysis. There are a large number of SNP and InDel loci widely distributed in the human genome, most of which have a high minor allele frequency in the population, and mutations occurring at these loci are usually not the main pathogenic factors. Most disease-associated mutations occur in the coding region or splice site region. Therefore, harmful mutations were filtered according to the following criteria to narrow the range of susceptibility gene screening: (1) the mutation located in the region of an exon or splice site, (2). nonsynonymous and frameshift mutations and gain or loss of a termination codon, (3) allele frequencies of the mutated loci in the database <1%, and (4) heterozygous mutations in patients’ samples and no mutation in the healthy sample (No. 4). After multiple screening conditions, a total of 12 SNV mutation sites and 9 InDels mutation sites were identified as candidate susceptibility genes.

Further, the influence of *SAMD9L* expression levels on the survival of 631 patients with gastric cancer patients was analyzed using a geographic database. The results show that patients with higher *SAMD9L* expression levels had better survival status, suggesting that *SAMD9L* may be related to the occurrence and development of gastric cancer. Unfortunately, RNA-seq is now very difficult to perform due to the lack of the tumor tissue (patients are not alive). We found that *SAMD9L* was low expressed in gastric cancer tissues compared with normal tissues in the TCGA database. Therefore, the *SAMD9L* p.T832fs mutation site may represent a susceptibility factor of this gastric cancer family, which is consistent with literature reports and bioinformatics analysis,^28^ and we performed analyses of cell functions.

*SAMD9L* is frequently mutated and inactivated in hepatocellular carcinoma, leading to a decrease in its expression levels and an increase in cell proliferation through accelerated cell cycle progression.^25^ These studies show that *SAMD9L* plays an important role in human malignant tumors and other diseases, although its role in the occurrence and development of gastric cancer is unknown.

Therefore, the cellular function of the *SAMD9L* gene was verified in vitro. *SAMD9L* was knocked down in SGC-7901 cells using the specific siRNA and shRNA. Inhibition of *SAMD9L* expression enhanced the proliferation, colony formation, migration and invasion of SGC-7901 cells, and rescued sorafenib-induced apoptosis. These results further verify the inhibitory effect of *SAMD9L* on cell proliferation. Furthermore, Kaplan–Meier patient survival was positively correlated with the expression level of *SAMD9L*, supporting that *SAMD9L* is a tumor suppressor. Here we show that a mutation of *SAMD9L* p.T832FS occurred in one allele of patients with gastric cancer and indirectly caused truncation mutation, which may lead to structural and functional abnormalities of SAMD9L protein. Since we have not performed any experiments on structural impact, this conclusion needs further verification. In addition, the length of *SAMD9L* gene coding sequence is 4752 bp, and generally, the length of the inserted fragment is less than 3000 bp. Considering that the constructed wild-type plasmid is too large, the transfecting efficiency of the plasmid is too low, the overexpression efficiency is too low, and even the target gene cannot be normally expressed in the cell. Thus, the study did not lead to further studies of wild-type *SAMD9L*. Furthermore, it is necessary to further study the effect of *SAMD9L* overexpression on gastric cancer cells through advanced methods to improve the efficiency of gene expression.

To conclude, these results indicate that the *SAMD9L* gene exerted inhibitory effects on cell proliferation, migration, and invasion. The present study demonstrates for the first time to our knowledge that the *SAMD9L* p.T832FS mutation represents a potential genetic susceptibility factor and tumor suppressor of FIGC, which may provide a valuable marker for early clinical diagnosis and treatment of FIGC.

## Figures and Tables

**Table 1.** Primers Sequences for PCR

Primer	Forward Primer/5′-3′	Reverse Primer/5′-3′
*LAMB1*	AACCTTACCA TTTCGGCAGC	TAGGACCCGAAGACACCAAA T
*EFCAB1*	GA TGTCCAAGCACCAAACCC	TGTCTTTGACGTTGTTTGTCTGT
*HSPG2*	A TGGCTGCTGACCTTGTTCG	GGCTGGATGTGAGAAAGAGTGTA

**Table 2.** Primer Sequence for qRT-PCR

Primer	Forward Primer/5′-3′	Reverse Primer/5′-3′
*SAMD9L*	ACTCTGACACACCCTCAGAA	AGTCTCTCTCTGGAAA TGCAGG
*SAMD9L*-Mutant	AATTCACGCACCAA TGGCAC	GTTGCTTGGAA TTGGCCAGG
*GAPDH*	GTCAAGGCTGAGAACGGGAA	AAA TGAGCCCCAGCCTTCTC

**Table 3.** *SAMD9L* shRNA and siRNAs Targeting Sequences

Primer	Forward Primer/5′-3′	Reverse Primer/5′-3′
si-1857	CCACGGAAGUGGACAUUAATT	UUAAUGUCCACUUCCGUGGTT
si-2677	GGAGAAGAUUUCUACUCUUTT	AAGAGUAGAAAUCUUCUCCTT
si-4095	CCUGGGAACCUGAAAGCUUTT	AAGCUUUCAGGUUCCCAGGTT
shRNA	CATCGCTACATAGAACATTAT	

**Table 4.** Candidate Susceptibility Genes of Familial Gastric Cancer

Gene	Chr	Start	End	Ref	Alt	Func
*SAMD9L*	7	92762790	92762790	**-**	T	Frameshift
*KRT18*	12	53343131	53343131	**-**	A	Frameshift
*ODF1*	8	103573024	103573031	GCCCCTGC	**-**	Frameshift
*ODF1*	8	103573033	103573042	ACCCGTGCAG	**-**	Frameshift
*RP1L1*	8	10465391	10465391	C	**-**	Frameshift
*RP1L1*	8	10465394	10465434	GGACCTCCCCT	**-**	Frameshift
*ZNF527*	19	37879852	37879852	**-**	TGTG	Frameshift
*ZNF527*	19	37879855	37879855	T	**–**	Frameshift
*FGL1*	8	17726470	17726470	**-**	T	Frameshift
*D2HGDH*	2	242695306	242695306	C	T	Nonsynonymous
*ZNF239*	10	44053013	44053013	G	C	Nonsynonymous
*AKAP9*	7	91739463	91739463	T	C	Nonsynonymous
*LAMB1*	7	107576008	107576008	T	G	Nonsynonymous
*EFCAB1*	8	49643961	49643961	G	A	Stopgain
*CYP46A1*	14	100193034	100193034	G	T	Nonsynonymous
*CEP170B*	14	105349504	105349504	C	T	Nonsynonymous
*HSPG2*	1	22186133	22186133	T	C	Nonsynonymous
*GPER1*	7	1131378	1131378	C	T	Nonsynonymous
*PTF1A*	10	23481688	23481688	G	A	Nonsynonymous
*EXOSC8*	13	37580090	37580090	C	T	Nonsynonymous
*KIRREL2*	19	36349459	36349459	G	A	Nonsynonymous
